# Modeling of Interactions between the Zebrafish Hatching Enzyme ZHE1 and A Series of Metal Oxide Nanoparticles: Nano-QSAR and Causal Analysis of Inactivation Mechanisms

**DOI:** 10.3390/nano7100330

**Published:** 2017-10-16

**Authors:** Natalia Sizochenko, Danuta Leszczynska, Jerzy Leszczynski

**Affiliations:** 1 Interdisciplinary Center for Nanotoxicity, Department of Chemistry, Physics and Atmospheric Sciences, Jackson State University, Jackson, MS 39217, USA; jerzy@icnanotox.org; 2 Department of Civil and Environmental Engineering, Jackson State University, Jackson, MS 39217, USA; danuta@icnanotox.org

**Keywords:** molecular descriptors, liquid drop model, QSAR, toxicity, metal oxide nanoparticles, regression tree, zebrafish, causality

## Abstract

The quantitative relationships between the activity of zebrafish ZHE1 enzyme and a series of experimental and physicochemical features of 24 metal oxide nanoparticles were revealed. Vital characteristics of the nanoparticles’ structure were reflected using both experimental and theoretical descriptors. The developed quantitative structure–activity relationship model for nanoparticles (nano-QSAR) was capable of predicting the enzyme inactivation based on four descriptors: the hydrodynamic radius, mass density, the Wigner–Seitz radius, and the covalent index. The nano-QSAR model was calculated using the non-linear regression tree M5P algorithm. The developed model is characterized by high robustness R^2^_bagging_ = 0.90 and external predictivity Q^2^_EXT_ = 0.93. This model is in agreement with modern theories of aquatic toxicity. Dissolution and size-dependent characteristics are among the key driving forces for enzyme inactivation. It was proven that ZnO, CuO, Cr_2_O_3_, and NiO nanoparticles demonstrated strong inhibitory effects because of their solubility. The proposed approach could be used as a non-experimental alternative to animal testing. Additionally, methods of causal discovery were applied to shed light on the mechanisms and modes of action.

## 1. Introduction

The various aspects of nanotechnology have been advancing for over half a century and continue to grow rapidly [1,2,3]. An increased usage of nanomaterials results in their release into the environment, making it necessary to determine the hazardous effects of these materials [4]. Determining the toxicity of materials is one of the main steps in safe materials design [5]. The majority of current studies are focused on the investigation of the environmental safety of nano-sized materials and devices [6,7].

Every year, an enormous amount of money is spent in both academia and industry on testing new hypotheses and the research and development of new nanomaterials [8]. Various alternatives to animal testing have been proposed to overcome some of the drawbacks associated with animal experiments [9]. The idea of the 3Rs approach (reduction, refinement, and replacement) is suitable for the investigation of nanoparticles (NPs). Different strategies have been proposed to reduce the number of experiments, from the standardization of testing conditions and extrapolation to the application of computational techniques [5,10]. For instance, as an alternative to animal testing, zebrafish has been replaced by its most representative enzyme: the zebrafish hatching metalloprotease, ZHE1 [11,12]. In the current project, we have developed a computational model based on the in vitro data of ZHE1 inhibition.

Over the years, different computational methods have been developed, but the quantitative structure–activity/properties relationships (QSAR/QSPR) approach is the most time-effective one [13,14,15]. The fundamental assumption of QSAR modeling is that the features of molecular structure are responsible for physical or biological properties of the studied samples [16]. Each compound could be represented by a set of different numbers (known as “descriptors”) [17]. For this purpose, the features of each chemical structure are defined by different physical or mathematical concepts and then transformed into descriptors [17]. In the last ten years, more than fifty QSAR/QSPR models for nanoparticles (known as “nano-QSAR models”) have been published [18,19].

Unique properties of NPs are governed by different characteristics related to the specific organization of NP (release of ions from a surface, huge contact area, aggregation effects, etc.). Therefore, researchers usually utilize experimental properties as descriptors: size, volume concentration, solubility, zeta potential, Brunauer–Emmett–Teller (BET) measurements (shape, surface area), reactivity, etc. [20,21,22]. Experimentally-based descriptors have a clear interpretation, which makes them a very valuable class of NP descriptors. However, experimental parameters can contain systematic or chance error, as the quality of measurements greatly depends on the equipment and the skills of the researcher [17]. This makes these descriptors applicable only for representing co-measured data (data obtained in the same laboratory).

As many existing models successfully combine simple descriptors with experimental data, we suggested that a successful depiction of the properties or mechanisms of action would become possible by means of a hierarchical combination of descriptors. The main idea of the hierarchical approach is a simultaneous use of descriptors that reflect the NPs’ structure at different levels of the organization of molecules: from ions to aggregates [23].

The objective of the current paper is the development of a theoretical 3R model that provides a safety assessment of the nanoparticles towards zebrafish. To reach this goal, a combination of theoretical descriptors with experimental parameters was applied for nano-QSAR modeling. As it has recently been pointed out, nanomaterials can demonstrate non-linear changes in toxicity. To address this problem in nano-QSAR modeling, a non-linear M5P tree approach was used. The general idea of the technique that was used is the creation of a conventional decision tree combined with linear regression functions [24]. Additionally, causal relationships between the activity and the descriptors of the developed model were analyzed.

## 2. Results

The developed nano-QSAR model has high statistical characteristics: R^2^_adj_ = 0.85 and RMSE = 0.062 for the training set, R^2^_adj_ = 0.69 and RMSE = 0.176 for the validation (bagging) set, R^2^_adj_ = 0.80 and RMSE = 0.112 for the external validation (test set). Prediction for Cr_2_O_3_ in the training set was outside of the three-sigma deviation for the developed model (outlier).

**Figure 1:**
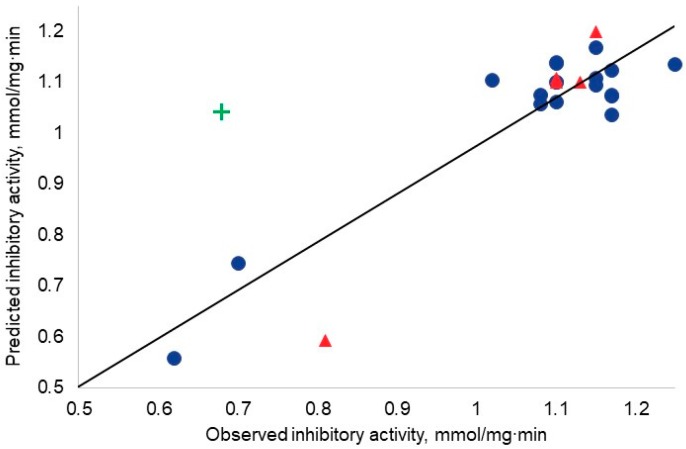
Observed–predicted diagram. Blue dots represent the training set; red triangles represent the test set; green cross represent the outlier.

The developed model to be discussed is non-linear. However, for the readers’ convenience, the observed–predicted diagram is presented in a classical way (in Figure 1). The straight line represents perfect agreement between the experimental and the calculated values.

**Figure 2:**
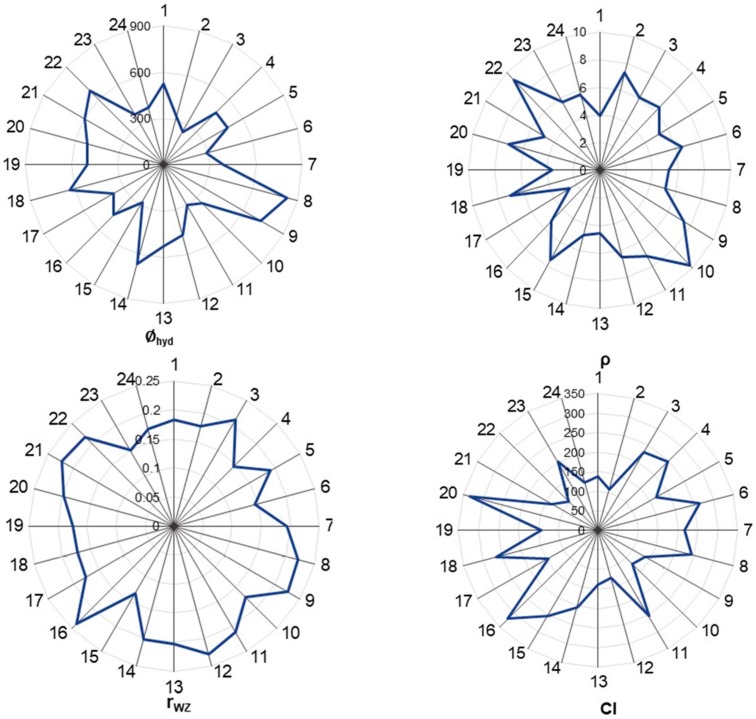
Radar plot of the distribution of used descriptors.

The developed model contains four descriptors: the hydrodynamic size (∅_hyd_), density (ρ), the Wigner–Seitz radius (*r*_wz_), and the covalent index (*CI*). In Figure 2, the distributions of values for the descriptors are represented using diagrams. QSAR methodology assumes that the structural variation causes the variation of physical or biological properties; therefore, adequate distribution of properties (descriptors) within the training/test set is crucial. As one can see from Figure 2, the distributions of selected descriptors varied considerably.

**Figure 3:**
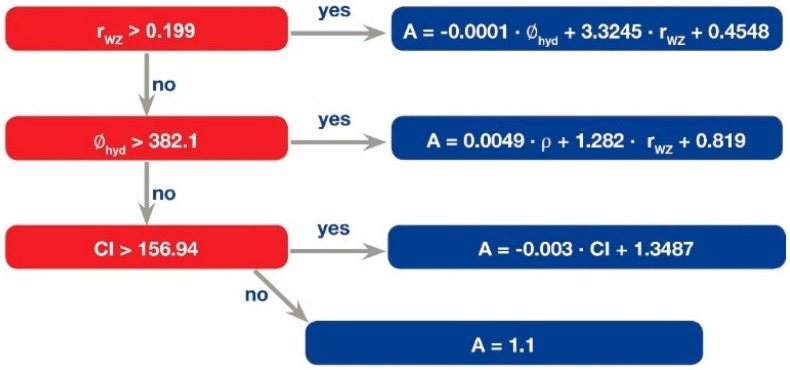
M5P regression tree.

The nano-QSAR model is presented in Figure 3. The developed non-linear model contains three nodes. The first node (group 1) covered ~42% of the training set (eight NPs): Ni_2_O_3_, Mn_2_O_3_, In_2_O_3_, Co_3_O_4_, Fe_3_O_4_, Y_2_O_3_, Gd_2_O_3_, La_2_O_3_. Similarly, the second node (group 2) covered ~31.5% (six NPs): Fe_2_O_3_, CoO, TiO_2_, WO_3_, Al_2_O_3_, SnO_2_. The third node (group 3) covered ~16% (three NPs): Cr_2_O_3_, ZnO, CuO. The remaining nanoparticles (group 4, ~10.5%, two NPs) were not included in the three main nodes: CeO_2_, HfO_2_. The percentage of involvement for each descriptor for all nodes (relative importance, RI) is presented in Table 1.

In order to evaluate the causality of the developed nano-QSAR model, a probability of causal relationships between toxicity and selected descriptors was measured. Direct and clear cause–effect relationships were found for the hydrodynamic size and toxicity with *p* = 0.05 in the first node (the Wigner–Seitz radius) and *p* < 0.001 for the remaining nodes.

## 3. Discussion

The percentage of relative importance of a descriptor reflects its mean influence. One of the most important advantages of non-linear models (such as decision tree-based models) is their potential to simultaneously describe different mechanisms of action. Each individual node (“leaf”) in a tree could be applied only to a specific group of nanoparticles. It is very critical for a numerical representation of nanoparticles, since different types of nanoparticles can affect certain targets (enzymes, cells, etc.) in opposite ways. As was mentioned in the introduction, the mechanisms of a nanoparticle’s action could vary as solubility, aggregates formation, and surface charge change.

Based on the above, low overall relative influence (8.2%) for the covalent index does not mean that it has low impact on the nanoparticle’s action. Group 4 is driven by the *CI* (Figure 3); therefore, the *CI* reflects only one mechanism of action (100%). It is critical to note that nanoparticles that were grouped by the *CI* (Figure 3, group 4)—namely ZnO and CuO in the training set and NiO in the validation set—demonstrated the highest ZHE1 inactivation.

**Figure 4:**
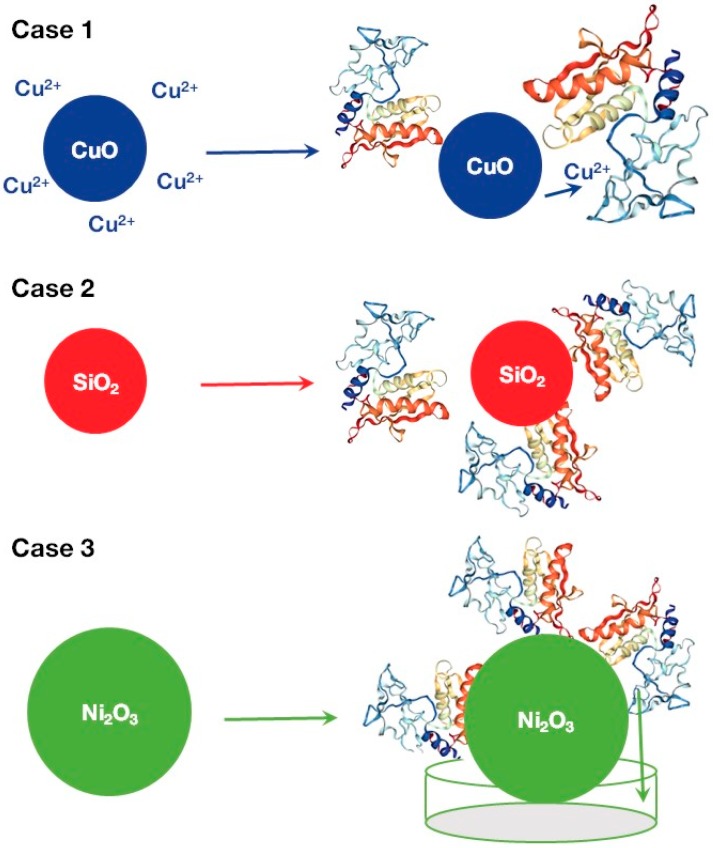
Schematic representation of mechanisms of the zebrafish hatching metalloprotease (ZHE1) inhibition induced by metal oxide nanoparticles.

The overall schematic representation of suggested toxicity mechanisms is outlined in Figure 4. The toxicity of nanoparticles could be related to a two-step process. At the first step, ions are released from the nanoparticle’s surface through the dissolution mechanism (Figure 4, Case 1). The likelihood of releasing depends on the environmental conditions and on the general trends of dissolution of the selected nanoparticle. Next, released metal ions interact with metal-sensitive binding sites in the active center of the zebrafish hatching enzyme, ZHE1 [11].

ZHE1 is a zinc metalloprotease. Generally, different divalent metal ions can replace the zinc ion in the enzyme’s binding site. However, binding affinity differs among a series of metal ions. For instance, Holt et al. demonstrated that the binding affinity of metals to metallothionein changed in the order Zn^2+^ < Cd^2+^ < Cu^2+^ < Hg^2+^ [25]. It was found that thermolysin activity increased when cobalt was present [26]. Replacement of Zn^2+^ ion by Cu^2+^ or Co^2+^ in the catalytic center of astacin increased enzymatic activity; inclusion of Ni^2+^ or Hg^2+^ inactivated the enzyme [27]. Similar results were obtained recently: metal substitution of Zn^2+^ by Co^2+^ or Mn^2+^ in dipeptidyl peptidase maintained catalytic activity [28]. On the contrary, Cu^2+^ that substituted dipeptidyl peptidase could not restore the catalytic activity. These findings were supported by the analysis of coordination geometries: Cu^2+^ coordination geometry is very rigid, while the coordination geometries of Zn^2+^, Co^2+^, and Mn^2+^ are flexible [25]. Moreover, even Zn^2+^ overdose can alter metalloprotease activity: *Burkholderia cenocepacia* ZmpA zinc metalloprotease activity was lowered by an excess of Zn^2+^ [29].

As it was pointed out by Lin et al., the ionic radius of metal ions, associated ion-ligand coordination chemistry, and binding constants could determine substitution in the enzyme center [12]. Based on the above, the theoretical descriptor *CI* simultaneously reflects the properties of released metal ions (charge and electronegativity) and interactions with the metal-binding amino acids in the enzyme center. Using numerical analysis, polynomial regression for the covalent index and the enzymatic activity of ZnO, CuO, and NiO (R^2^ = 0.85) were identified. Therefore, the descriptor *CI* represents an attractive theoretical parameter that reflects mechanisms of action of released metal ions.

As one can see from Table 2, an influence of the hydrodynamic size and the Wigner–Seitz radius have the highest impacts and contribute equally to the enzyme inhibition. We suggest that the hydrodynamic size and the Wigner–Seitz radius represent similar—or possibly the same—mechanism of action (Figure 4, Case 2 and Case 3). For instance, nanoparticles that were characterized by higher values of the Wigner–Seitz radius (*r*_wz_ > 0.196, combination of area 1 and area 2 in Figure 3) demonstrated simple linear relationships between enzymatic activity and the hydrodynamic size (Pearson’s correlation coefficient *r* = −0.91).

With a high probability, the Wigner–Seitz radius indirectly reflects the fraction of molecules on the nanoparticle’s surface, which could be responsible for toxicity or inhibitory activity (Figure 4, Case 2). In general, the high influence of nanoparticles is caused by their large specific surface area (Figure 4, Case 2). Indeed, metal oxides with higher values of the Wigner–Seitz radius usually have a smaller surface-to-volume ratio (when sizes of studied nanoparticles are the same). A smaller surface-to-volume ratio leads to lower activity. This is in agreement with the developed model (see group 1 and group 2, in Figure 2). The groups of nanoparticles with *r*_wz_ > 0.196 demonstrated lower activity.

Moreover, as it was demonstrated in our previous contribution, the “liquid drop” model (LDM)-based Wigner–Seitz radius (*r*_w_) and mass density (ρ) are mutually-related descriptors [30]. The Wigner–Seitz radius (*r*_w_) and mass density (ρ) are not directly correlated to each other in statistical terms, but the relationship between ρ and *r*_w_ has a straightforward mechanistic interpretation and can be deducted from the basic LDM equation (Equation (1)).

As it was mentioned, the hydrodynamic size and the Wigner–Seitz radius are also related to the third possible mechanism (Figure 4, Case 3). Another important process represents the self-assembly of nanoparticles in large aggregates or agglomerates in a culture medium (Figure 4, Case 3). Large nanoaggregates are not able to reach the binding site of the enzyme. If these nanoaggregates are poorly soluble, they do not release substantial amounts of metal ions. As a result, metal ions are not capable of reaching the binding site of the enzyme. In general, poorly soluble or non-soluble nanoparticles could inactivate enzymes only blocking outer surfaces. In other words, it is possible that certain metal oxide nanoparticles adsorb the target enzyme. However, at the same time, larger aggregates reach enzymes (or cells) faster than smaller particles due to gravity sedimentation. For example, Ni_2_O_3_ or Fe_3_O_4_ (Table 1, and Figure 4, Case 3) were characterized by a large hydrodynamic diameter (665.8 and 831.7 nm, respectively) and demonstrated higher enzyme inhibition than smaller nanoparticles aggregated from group 1 (In_2_O_3_, Co_3_O_4_, and La_2_O_3_, ∅_hyd_ < 450).

Summarizing the data presented in Figure 4, one can conclude that the release of metal ions from the surface of NP could cause their interaction with metal-sensitive binding sites in the active center of the enzyme (Case 1, Figure 4); the high influence of nanoparticles is caused by their large specific surface area (Case 2, Figure 4); aggregation can positively or negatively influence the dynamics of metal ion accumulation (Case 3, Figure 4).

## 4. Materials and Methods

### 4.1. Biological Activity Data

The enzymatic activity of metalloprotease zebrafish hatching enzyme ZHE1 (abiotic enzyme assay) for 24 nano-sized silica- and metal oxides was analyzed and all original experimental data were taken from literature [12]. Chemical structures, enzymatic activity, zeta-potentials, and hydrodynamic sizes (as measured in Holtfreter’s medium, pH 7.0) are given in Table 1.

### 4.2. Theoretical Descriptors

A simplified theoretical model for NPs representation—the “liquid drop” model (LDM)—has recently been introduced [24,31,32]. Geometrical transformations help calculate various size-dependent theoretical descriptors. In LDM, an NP is represented as a spherical drop. Basic elements (e.g., single atoms, molecules, or NPs in aggregate) are densely packed, and the density of a whole NP is equal to mass density. The general idea of the LDM approach lies in the assumption that each separate element is characterized by a radius of minimal interaction—the Wigner–Seitz radius (*r*_w_):

(1) $$r_{wz}={\left(\frac{3M}{4\pi \rho }\right)}^{\frac{1}{3}},$$

where *M* is molecular weight and ρ is mass density.

The Wigner–Seitz radius represents imaginary basic elements (such as molecules, crystal units, or NPs in aggregate) inside nanoparticles. Depending on the type of basic elements, basic equation can be modified.

It is widely known that ionic forms of metals are more active because of stronger binding of ions to biomolecules [33]. In this connection, several ionic characteristics were developed for QSAR modeling [24,33]. In the current study, the covalent index (*CI*) was utilized to describe a metal ion’s affinity:

(2) $$CI=\chi^{2}r$$

where *χ* is electronegativity and *r* is the Pauling radius.

### 4.3. QSAR Modeling

The validation of the model was performed by splitting the experimental data into the training, bagging, and external validation sets. The training set covers ~80% of the initial dataset; the external validation set covers the remaining ~20%; the bagging set covers ~60% of the training set. Bagging was used to reduce overfitting. This technique is close to the well-known “leave-one-out” validation in classical regression modeling. During bagging, predictions composed of *X* random models fit to bootstrap samples. For the readers interested in a detailed description of the bagging technique, Breiman’s original paper is strongly recommended [34].

NPs from the training set were structurally diverse to cover the whole descriptors space of the overall data set. Metal oxides were homogeneously split between the training and the external validation sets by ranging activity and covering all types of oxides (MeO, Me_2_O_3_, MeO_2_) for each set (Table 2).

Nano-QSAR tasks were carried out using the M5P approach [35]. The models were developed using the Weka software package (version 3.6) and its integration workflow plan for KNIME 2.11. M5P methodology combines a conventional decision tree technique with the possibility of including linear regression functions at the nodes. At the first step, a tree model was generated and specific tree nodes were identified. At the next step, a multivariate linear model was constructed for the training samples at each node obtained at the first step. The relative importance (RI) of variable score for every descriptor included in the model was calculated. The importance indicated what share a certain descriptor contributed to the modeled property. To reach this goal, the mean deviation of correct classifications was computed. In our previous contribution, we successfully demonstrated application of the M5P technique incorporated in KNIME 2.11 for the nano-QSAR modeling [32].

The statistical fit of the model was assessed by the adjusted determination coefficient R^2^_adj_, the bagging correlation coefficient Q^2^_bagging_, and the external validation coefficient Q^2^_EXT_. Root-mean-square error RMSE was calculated for all sets.

(3) $$R^{2}=\frac{\sum_{i}{\left({\hat{y}}_{i}-\bar{y}\right)}^{2}}{\sum_{i}{\left(y_{i}-\bar{y}\right)}^{2}}$$

where $y_{i\;}$ represents the original data values, ${\hat{y}}_{i}$ the modeled values, and $\bar{y}$ is the estimate of the mean.

(4) $$RMSE=\sqrt{\frac{\sum_{i}{\left({\hat{y}}_{i}-y\right)}^{2}}{m-1}}$$

where *m* is the number of data values.

### 4.4. Causal Relationships Modeling

A large number of publications related to QSAR/QSPR modeling contain a logical fallacy due to the fact that causality was incorrectly assigned to the variables that were only correlated (e.g., see discussion in our previous papers [30,33]). To avoid the logical fallacy, causal discovery methods could be beneficial for simultaneous application with QSAR methods. When the causal relationship is established, prevention of potential hazardous effects becomes possible.

The relationship between two sets of events, where one set of events (the effects) is a direct consequence of another set of events (the causes), is called “causality”. In the current contribution, the Peter Spirtes and Clark Glymour (PC) algorithm was applied to estimate causal relationships between the descriptors and toxicity towards ZHE1 [36].

The PC algorithm states that at the first step all variables are connected within a complete undirected graph. Next, conditional independence tests allow non-important edges to be deleted. The maximum likelihood function over all variables has been maximized, and the null hypothesis based on the assumption that the population covariance matrix is equal to the estimated covariance matrix has been introduced. The results could be presented as a function of the free model parameters [36].

## 5. Conclusions

Understanding the factors that play a prevailing role in the activity of nanoparticles is crucial for establishing the mechanism of their toxicity. In this study, we have analyzed clearly interpretable factors and built a quantitative model that describes the relationships between the structural characteristics of metal oxide nanoparticles and their enzymatic activity. It was demonstrated that the combination of experimental parameters and theoretical descriptors could be beneficial for a representation of structural features of nano-sized metal oxides. The nano-QSAR model developed here reliably predicts the activity of the studied nanoparticles towards the zebrafish hatching enzyme. The interpretation of the descriptors included in the model is strictly related to possible mechanisms of the nanoparticles’ action. It was found that the theoretical Wigner–Seitz radius and the experimental hydrodynamic radius have similar contributions to inhibitory effect and represent non-specific interactions between the enzyme and the nanoparticle. It was demonstrated that specific surface and metal ion release are important for the evaluation of metal oxide nanoparticles’ toxicity.

## Figures and Tables

**Table 1:** List of descriptors and their relative importance (RI, %) in quantitative structure–activity relationship (QSAR) model. LDM: “liquid drop” model.

Descriptor	Symbol	Descriptor Type	RI, %
hydrodynamic size	∅_hyd_	experimental	37.8
density	ρ	LDM	16.2
Wigner–Seitz radius	*r*_wz_	LDM	37.8
covalent index	*CI*	ionic	8.2

**Table 2:** Experimental data and theoretical descriptors. NP: nanoparticle.

NP	Hydrodynamic Size (∅hyd), nm	Density (ρ), g/sm^3^	Wigner–Seitz Radius (*r*_wz_), a.u.	Covalent Index (*CI*)	Enzyme Activity (*A*), mmol/mg∙min
Al_2_O_3_	524.8	3.96	0.183	138.68	1.17
**CeO_2_**	321.3	7.30	0.178	109.13	1.10
Co_3_O_4_	247.6	6.07	0.212	229.74	1.25
CoO	378.3	6.40	0.144	247.41	1.17
Cr_2_O_3_	478.5	5.21	0.191	168.09	0.68
CuO	289.5	6.45	0.143	263.53	0.62
Fe_2_O_3_	385.2	5.25	0.194	216.00	1.15
Fe_3_O_4_	831.7	5.20	0.220	241.12	1.02
Gd_2_O_3_	726.7	7.41	0.227	134.64	1.10
HfO_2_	349.9	9.68	0.173	119.99	1.10
In_2_O_3_	303.2	7.18	0.210	253.47	1.17
La_2_O_3_	471.2	6.51	0.229	124.87	1.15
Mn_2_O_3_	525.9	4.55	0.202	139.35	1.08
Ni_2_O_3_	665.8	4.83	0.201	204.29	1.08
**NiO**	277.5	7.45	0.134	251.72	0.81
**Sb_2_O_3_**	459.9	5.19	0.238	319.39	1.15
SiO_2_	374.9	2.65	0.176	144.40	1.10
SnO_2_	635.0	7.01	0.173	265.07	1.17
TiO_2_	497.0	3.60	0.174	143.48	1.10
WO_3_	511.9	7.20	0.197	334.18	1.15
Y_2_O_3_	594.5	4.84	0.223	133.96	1.10
**Yb_2_O_3_**	682.6	9.25	0.217	105.03	1.10
ZnO	379	5.70	0.150	201.47	0.70
**ZrO_2_**	384.4	5.68	0.173	127.36	1.13
Control value	-	-	-	-	1.25

Nanoparticles used for external validation set are marked in bold.
